# Anxiety Symptoms in Hypothyroidism: A Case for Causation or Co-Occurrence?

**DOI:** 10.7759/cureus.77814

**Published:** 2025-01-22

**Authors:** Eleftheria Dampa

**Affiliations:** 1 Internal Medicine, Kilkis General Hospital, Kilkis, GRC

**Keywords:** anxiety, anxiety symptoms, gad, hypothyroidism, levothyroxine, thyroid function tests

## Abstract

Hypothyroidism, also known as an underactive thyroid, is an endocrine condition in which the thyroid gland does not produce enough thyroid hormones to meet the body’s needs. Hypothyroidism can develop slowly over time and cause a variety of symptoms, including fatigue, muscle weakness and stiffness, cold intolerance, brain fog, anxiety, limb numbness or tingling sensation, and palpitations. The following case describes a 32-year-old female who presented with anxiety-like symptoms closely resembling the classic symptomatology of hypothyroidism. These symptoms led to a 10-month misdiagnosis of generalized anxiety disorder. The patient had never been tested for hypothyroidism, nor had she undergone a thyroid panel. Following additional assessments, the patient was diagnosed with hypothyroidism, which emphasizes the necessity of conducting a comprehensive evaluation of the endocrine system and potential root causes in individuals exhibiting anxiety-like symptoms. This case report highlights the importance of conducting thorough medical and endocrine evaluations in patients with anxiety-like symptoms to prevent misdiagnosis, avoid unnecessary treatments, and ensure proper care.

## Introduction

Hypothyroidism is a common clinical condition characterized by inadequate production of thyroid hormones by the thyroid gland. Hypothyroidism can be divided into three categories: primary, where the thyroid gland itself is unable to produce sufficient T3 and T4 thyroid hormones; secondary, where the pituitary gland fails to secrete adequate thyroid-stimulating hormone (TSH); and tertiary, where there is hypothalamic dysfunction that leads to reduced secretion of thyrotropin-releasing hormone (TRH). In iodine-sufficient regions, Hashimoto’s thyroiditis - an autoimmune disorder in which the immune system mistakenly attacks the thyroid gland, leading to chronic inflammation and thyroid dysfunction over time - is the most common cause of hypothyroidism [1]. The standard practice for diagnosing hypothyroidism involves conducting thyroid function tests to assess thyroid function, with a primary focus on measuring TSH [2]. Evaluating TSH is the most reliable method for diagnosing hypothyroidism, as elevated TSH levels indicate the condition [3].

Anxiety disorders are a group of mental health conditions characterized by excessive and persistent feelings of fear, worry, and/or apprehension. These feelings can interfere with an individual’s daily activities and may be out of proportion to the actual situation or threat. Difficulty sleeping, sweating and trembling, restlessness, palpitations, tiredness, and irritability are some of the symptoms that might be present [4]. Regrettably, these symptoms and manifestations can closely resemble those of hypothyroidism, making accurate diagnosis and treatment more challenging. Anxiety itself can complicate the management of hypothyroidism. As the diagnosis of hypothyroidism relies on blood tests and a comprehensive thyroid panel, individuals may remain undiagnosed throughout their lives or mistakenly attribute their symptoms solely to anxiety. However, hypothyroidism could be responsible for the majority of these symptoms if not properly excluded first. Therefore, it is essential to rule out any underlying endocrine conditions before assigning a psychiatric diagnosis. The following case report presents a distinct situation where hypothyroidism presented with symptoms mimicking an anxiety disorder thus leading to a misdiagnosis. This draws attention to the absolute necessity of first considering and ruling out endocrine conditions in patients with symptoms resembling anxiety.

## Case presentation

A 32-year-old female patient presented with complaints of generalized muscle tension, lethargy, and persistent fatigue. She denied any history of trauma, fever, or weight changes. The patient reported significant difficulty falling asleep and frequent restlessness, attributing these issues to insufficient sleep. She denied the use of medications or recreational drugs and had no history of significant personal medical conditions or surgeries. Her family history was unremarkable, with no reported cases of endocrine, psychiatric, or autoimmune disorders. She described feeling tense throughout the day, often experiencing headaches and an ongoing sense of unease. Concentration was another significant concern, which she attributed to procrastination and brain fog; she reported experiencing continuous brain fog, fluctuating moods, and persistent difficulties with focusing. The patient admitted to being unable to control her worrying, occasionally losing her patience with others, and feeling emotionally drained. Although she consistently felt fatigued, she also described a sense of hypervigilance, which left her feeling both physically and mentally exhausted. Her sleep disturbances were particularly distressing, as she described being unable to fall asleep easily and frequently waking up during the night for no apparent reason. She reported a lifelong tendency toward excessive sleepiness, which others often criticized by labeling her as lazy, causing her significant feelings of shame and embarrassment. She also reported a history of dry skin, which she had managed with over-the-counter moisturizers. When the patient initially sought medical help for her symptoms, she consulted an external private physician. During the visit, her vital signs were found to be within normal limits. She was prescribed 50 mg of sertraline daily and advised to reduce her stress levels. However, no psychiatric assessments or additional evaluations were conducted by the private physician at that time.

Two months later, she visited the internal medicine outpatient department at the hospital, seeking a second opinion due to persistent symptoms and frustration over the lack of improvement despite being on sertraline. She reported experiencing persistent fatigue, cognitive impairment commonly described as “brain fog,” and sleep disturbances, expressing concerns that the prescribed medication had not alleviated her symptoms and may have exacerbated them. She reported feeling drowsy, nauseated, and restless while on sertraline. A thorough physical and neurological examination was conducted, with normal findings. Her ECG was normal with no pathological findings. Her lungs on auscultations were normal with no pathological hearing sounds, and her cardiac auscultation revealed normal heart sounds without any murmurs. However, her facial appearance was puffy, particularly around the eyes, and her face appeared slightly erythematous. Her extremities felt cold to the touch, and she reported feeling cold most of the time. The skin on her hands was dry and flaky, and her nails were brittle and prone to breaking, but no scars, cuts, or blisters were visible. Fine hand and peripheral tremors were observed. No signs of exophthalmos were present. Examination of her thyroid revealed a gland of normal size on palpation with no bruit on auscultation. Her vital signs showed a blood pressure of 128/72 mmHg, a heart rate of 90 beats per minute, a temperature of 36.5°C, oxygen saturation at 98% on room air, and a BMI of 23.3 kg/m². Upon further questioning, she mentioned that her menstrual cycles were normal but had always been heavy. A psychiatric assessment was conducted using a hospital-based Generalized Anxiety Disorder-7 (GAD-7) screening tool (Table 1). The total score was 7, reflecting mild anxiety that requires only symptom monitoring and routine follow-ups.

**Table 1:** Generalized Anxiety Disorder-7 screening chart Total score = 7 (mild anxiety) GAD-7 total score for the seven items ranges from 0 to 21 0-4: minimal anxiety; 5-9: mild anxiety; 10-14: moderate anxiety; 15-21 severe anxiety

How often has the patient been bothered by the following symptoms over the past 2 weeks	Not at all (0 points)	Several days (1 point)	More than half the days (2 points)	Nearly every day (3 points)
Feeling nervous, anxious, or on edge	-	X	-	-
Not being able to stop or control worrying	-	X	-	-
Worrying too much about different things	-	X	-	-
Trouble relaxing	-	X	-	-
Being so restless that it’s hard to sit still	X	-	-	-
Becoming easily annoyed or irritable	-	-	X	-
Feeling afraid as if something awful might happen	-	X	-	-

Laboratory tests included a complete blood count (CBC), biochemistry blood tests, a urine pregnancy test, and thyroid function tests. The pregnancy test was negative, and the CBC revealed no abnormalities. The thyroid function tests exhibited high TSH levels and low T4 levels, thereby confirming the diagnosis of hypothyroidism. The results of the thyroid function tests are summarized in Table 2, while the findings from the complete blood count (CBC) are detailed in Table 3. Additionally, the results of the biochemistry tests are provided in Table 4.

**Table 2:** Thyroid function tests TSH, thyroid stimulating hormone; T3, triiodothyronine; T4, thyroxin

Test	Patient value	Normal range
TSH (mlU/L)	12.3 mIU/L	0.5 - 5.0 mIU/L
Free T3 (pmol/L)	3.2 pmol/L	2.0 - 7.0 pmol/L
Free T4 (pmol/L)	5.1 pmol/L	10.3 - 24.5 pmol/L

**Table 3:** Complete blood count (CBC)

Test	Patient value	Normal range
HCT (%)	39%	35 - 47.4
HB (g/dL)	14.8	12 - 16
RBC Count (M/μL)	4.68	4.20 - 5.50
MCV (fL)	83.8	78.0 - 98.0
MCH (pg)	28.1	27.0 - 31.0
MCHC (gr/dL)	33.5	32.0 - 36.0
WBC (K/μL)	8.6	4.2 - 11.5
NEUT (%)	57.9	40.0 - 70.0
LYM (%)	34.5	19.0 - 48.0
MONO (%)	7.6	3.4 - 9.0
PLT (K/μL)	250	150 - 450

**Table 4:** Biochemistry lab tests

Test	Patient value	Normal range
Blood Glucose (mg/dL)	84	55 - 120
Urea (mg/dL)	28.0	20.0 - 50.0
Creatinine (mg/dL)	0.66	0.50 - 1.10
HDL (mg/dL)	64	35 - 80
LDL (U/L)	130	<248
Potassium (K) (mEq/L)	3.9	3.5 - 5.1
Sodium (Na) (mEq/L)	141.2	135 - 145
CPK (U/L)	54	26 - 192
Triglycerides (mg/dL)	60	40-140
CRP (mg/L)	0.96	< 5.00

The patient was subsequently referred to an endocrinologist for further evaluation and treatment. She was prescribed levothyroxine at a dose of 100 mcg, to be taken in the morning on an empty stomach, approximately 20 minutes before breakfast. Additionally, sertraline was gradually reduced to a dose of 25 mg over two weeks before being discontinued entirely. During a follow-up visit two months later, the patient reported significant improvement and a positive response to the treatment. She described experiencing immediate mental clarity, with her brain fog markedly improving by the second week of therapy. By the fourth week, her anxiety-like symptoms had almost completely resolved. The patient reported a significant improvement in her daily energy levels, accompanied by an enhanced ability to fall asleep promptly and maintain uninterrupted sleep throughout the night. She described her life as having undergone a “complete transformation.” Additionally, she noted marked progress in her capacity to concentrate effectively and accomplish tasks, successfully overcoming years of chronic procrastination. By the third month of levothyroxine therapy, her thyroid function tests showed that both TSH and T4 levels had normalized, albeit at the higher end of the reference range. At this point, the levothyroxine dose was reduced to 50 mcg. By the fourth month, her TSH and T4 levels were firmly within the normal range, and her dose was further tapered to 25 mcg. Regular monitoring, including monthly assessments of thyroid function, was instituted to maintain optimal hormone levels. This gradual reduction in levothyroxine dosage successfully maintained stable thyroid function while minimizing the risk of overtreatment.

## Discussion

When evaluating hypothyroidism as a primarily endocrine disorder, it is imperative to take into account the extensive spectrum of psychiatric manifestations it may present before attributing anxiety-like symptoms exclusively to a psychiatric diagnosis. These symptoms are not limited to anxiety-like presentations but may also include depression-like symptoms and cognitive impairments [5]. Research by Cooke et al. demonstrated a measurable reduction in the volume of the right hippocampus in individuals with adult-onset overt hypothyroidism [6]. As the right hippocampus plays a critical role in long-term and spatial memory in adults, this finding highlights the profound effect hypothyroidism can have on memory formation and information retrieval in a person. These cognitive impairments play a significant role in the phenomenon commonly referred to as “brain fog.” They are frequently misinterpreted as primary psychiatric disorders, which can result in incorrect diagnoses, delays in receiving proper care, and, in some instances, the use of unwarranted or ineffective treatments. A systematic review and meta-analysis [7] examining the association between autoimmune thyroiditis (AIT), a major cause of hypothyroidism, and anxiety disorders found that approximately 35.7% of individuals with AIT experience anxiety disorders compared to healthy individuals, emphasizing a notable link between thyroid dysfunction and anxiety. Another study conducted in India [8] assessed 100 patients diagnosed with hypothyroidism using the Hamilton Anxiety Rating Scale (HAM-A). The results [8] revealed that about 63% of the patients exhibited some degree of anxiety, with a higher prevalence observed in females (65.72%) compared to males (56.66%). Additionally, a nationwide registry study [9] conducted in Denmark explored the prevalence of psychiatric conditions before and after a hypothyroidism diagnosis. The study revealed that individuals with hypothyroidism were at a higher risk of being diagnosed with psychiatric disorders, including anxiety, and were more likely to receive treatment with antidepressants, antipsychotics, and anxiolytics both before and after their diagnosis [9].

Gamma-aminobutyric acid (GABA), the most important inhibitory neurotransmitter in the brain, can be affected by thyroid dysfunction. GABA plays a key role in modulating mood and cognition. In a study conducted by Liu et al., patients with hypothyroidism were found to have significantly decreased GABA+ levels in the medial prefrontal cortex (mPFC) compared to healthy controls [10]. After six months of treatment with levothyroxine (L-T4), a significant increase in GABA+ concentrations was observed, leading to the normalization of GABA+ levels in the brain [10]. This evidence suggests that reduced GABA+ levels could play a significant neurobiological role in the pathophysiology of hypothyroidism. Moreover, the effectiveness of levothyroxine in reversing cognitive impairments caused by hypothyroidism underscores its potential as an adjunctive therapy for managing affective disorders [10].

One key takeaway from this case is the necessity of including thyroid function tests in the diagnostic workup of patients presenting with persistent anxiety symptoms, particularly when these symptoms are accompanied by physical signs such as fatigue, cold intolerance, dry skin, and brittle nails. The lack of an initial endocrine evaluation in this patient highlights a critical gap in the diagnostic process, resulting in months of ineffective treatment with sertraline. The subsequent reduction and eventual cessation of sertraline following the initiation of levothyroxine therapy emphasize the potential to avoid unnecessary psychotropic medications when an underlying endocrine etiology is properly identified and addressed.

## Conclusions

This case highlights the critical interplay between endocrine and psychiatric disorders, emphasizing the need for a comprehensive and multidisciplinary diagnostic approach when patients present with anxiety-like symptoms. Hypothyroidism, though primarily an endocrine disorder, can exhibit psychiatric manifestations that closely mimic anxiety disorders, potentially leading to misdiagnosis and delayed treatment. The resolution of this patient’s symptoms following levothyroxine therapy underscores the importance of considering thyroid dysfunction as an underlying cause in patients with persistent anxiety, especially when accompanied by somatic symptoms such as fatigue, cold intolerance, and dry skin. This case serves as a valuable reminder that addressing the root cause, rather than treating symptoms in isolation, can result in substantial clinical improvement and prevent the unnecessary use of psychotropic medications. In clinical practice, it is essential to include thyroid function tests in the diagnostic assessment of patients exhibiting anxiety-like symptoms. This helps rule out a potentially treatable condition before arriving at a psychiatric diagnosis. Early identification and treatment of hypothyroidism can prevent prolonged patient distress, ensure timely management, and improve overall quality of life.
